# RNA Sequencing revealed differentially expressed genes functionally associated with immunity and tumor suppression during latent phase infection of a vv + MDV in chickens

**DOI:** 10.1038/s41598-019-50561-x

**Published:** 2019-10-02

**Authors:** Kunzhe Dong, Shuang Chang, Qingmei Xie, Peng Zhao, Huanmin Zhang

**Affiliations:** 1 USDA, Agricultural Research Service, Avian Disease and Oncology Laboratory, East Lansing, MI 48823 USA; 2 ORISE Fellow, USDA, Agriculture Research Service, Avian Disease and Oncology Laboratory, East Lansing, MI 48823 USA; 3 College of Veterinary Medicine, Shandong Agricultural University, Tai’an, Shandong 271018 China; 4 College of Animal Science, South China Agricultural University, Guangzhou, 510642 China

**Keywords:** Cancer genomics, Genomics

## Abstract

Very virulent plus Marek’s disease (MD) virus (vv + MDV) induces tumors in relatively resistant lines of chickens and early mortality in highly susceptible lines of chickens. The vv + MDV also triggers a series of cellular responses in both types of chickens. We challenged birds sampled from a highly inbred chicken line (line 6_3_) that is relatively resistant to MD and from another inbred line (line 7_2_) that is highly susceptible to MD with a vv + MDV. RNA-sequencing analysis was performed with samples extracted from spleen tissues taken at 10-day and 21-day post infection (dpi). A total of 64 and 106 differentially expressed genes was identified in response to the vv + MDV challenge at latent phase in the resistant and susceptible lines of chickens, respectively. Direct comparisons between samples of the two lines identified 90 and 126 differentially expressed genes for control and MDV challenged groups, respectively. The differentially expressed gene profiles illustrated that intensive defense responses were significantly induced by vv + MDV at 10 dpi and 21 dpi but with slight changes in the resistant line. In contrast, vv + MDV induced a measurable suppression of gene expression associated with host defense at 10 dpi but followed by an apparent activation of the defense response at 21 dpi in the susceptible line of chickens. The observed difference in gene expression between the two genetic lines of chickens in response to MDV challenge during the latent phase provided a piece of indirect evidence that time points for MDV reactivation differ between the genetic lines of chickens with different levels of genetic resistance to MD. Early MDV reactivation might be necessary and potent to host defense system readiness for damage control of tumorigenesis and disease progression, which consequently results in measurable differences in phenotypic characteristics including early mortality (8 to 20 dpi) and tumor incidence between the resistant and susceptible lines of chickens. Combining differential gene expression patterns with reported GO function terms and quantitative trait loci, a total of 27 top genes was selected as highly promising candidate genes for genetic resistance to MD. These genes are functionally involved with virus process (*F13A1* and *HSP90AB1*), immunity (*ABCB1LB*, *RGS5*, *C10ORF58*, *OSF*-*2*, *MMP7*, *CXCL12*, *GAL1*, *GAL2*, *GAL7*, *HVCN1*, *PDE4D*, *IL4I1*, *PARP9*, *EOMES*, *MPEG1*, *PDK4*, *CCLI10*, *K60* and *FST*), and tumor suppression (*ADAMTS2*, *LXN*, *ARRDC3*, *WNT7A*, *CLDN1* and *HPGD*). It is anticipated that these findings will facilitate advancement in the fundamental understanding on mechanisms of genetic resistance to MD. In addition, such advancement may also provide insights on tumor virus-induced tumorigenesis in general and help the research community recognize MD study may serve as a good model for oncology study involving tumor viruses.

## Introduction

Marek’s disease (MD) is a neoplastic disease of chickens caused by an oncogenic alpha-herpesvirus, commonly known as MD virus (MDV). MD is characterized by a variety of clinical signs, including immune suppression, polyneuritis, and notably, the formation of T-cell lymphomas that manifest as solid tumors^1^. MD has had being a major concern for the poultry industry worldwide since the 1900s^2^, which causes an economic loss as high or over $2 billion per year^3^ up to date resulted from condemnation, vaccination, and extra management measures necessary for the control. Hence, a better understanding on genomic mechanism underlying virus-induced tumorigenesis and progression would not only benefit the poultry industries, but also bring significant merits for human health in the foreseeable future.

Like other herpesviruses, MDV pathogenesis in chicken involves multiple phases including cytolytic and latent phases^4^. MDV initially enters an early cytolytic infection phase in B cells between 3- and 6-days post infection (dpi)^5^. The virus replication reaches peak at this stage^6^. Around 7 dpi, MDV-infected B cells transfer MDV to T cells reportedly through cell to cell contacts and the virus then quickly establishes latency between 7–10 dpi in the T cells^7,8^. During the latency phase, the MDV genome remains persistent in the host cells^4^. In susceptible chickens, a second cycle of cytolytic infection takes place at around 14 dpi as a result of virus reactivation, which causes inflammation and lymphoma formation around 21 dpi and onward^1^. MD regression has been reported and suggested to be affected by virus and host genotypes as well as subsequent immune responses^9–11^. During infection, MDV attempts to control or take advantage of host components to facilitate its replication. At the meantime, host mobilizes its innate immune system to battle against the viral infection and replication processes^1,4,12^. Therefore, the disease progression is clearly influenced by a complex set of interactions involving, at least, the viral genome and the host’s immune systems. To date, much efforts have been focused on advancing the understanding of the pathogen and its remarkable repertoire of virulence factors. Many viral genes have been extensively studied and documented, such as Meq and vIL-8, which are functionally involved in cell transformation, tumorigenesis and tumor malignance^13^. On the other hand, what host genes affect MD resistance is relatively far from clearly understood.

It is well documented that following MDV challenge, MD incidence occurs differently among different genetic lines of chickens due to varied susceptibility^14–18^. Striking examples include the highly inbred chicken lines 6_3_ and 7_2_, which were developed and maintained since 1939 at the USDA, Avian Disease and Oncology Laboratory (ADOL), East Lansing, Michigan, U.S.A. The lines 6_3_ and 7_2_ share a common major histocompatibility complex haplotype (B*2) but differ significantly in MD resistance^16,17^. When challenged with a partially attenuated very virulent plus strain of MDV (648A passage 40), up to 100% of the line 7_2_ chickens developed MD while over 90% of line 6_3_ chickens remained MD free 8 weeks post MDV inoculation^19^. These inbred lines of chickens are commonly considered as ideal resources for investigating the mechanisms of genetic resistance to MD and MD tumor progression^15,20^. Since the establishment of the genetic lines, a variety of differences in response to MDV infection between the two lines of chickens have been investigated, which include the aggregate number of target lymphocytes for virus infection and transformation^21,22^, expressed antigens on T-cell surface^23^, the number of infected lymphocytes during the early lytic phase of infection^24^, viral load during latent phase of infection^25,26^, the expression of Interferon (INF) genes^27^ and cytokine genes^26^. However, little is known about the difference of transcriptomic patterns in response to vv + MDV challenge at the latent and the late cytolytic phases between the resistant and susceptible lines of chickens, despite that extensive efforts have been put in to elucidate the gene expression in response to MDV challenge^3,12,28–36^. Furthermore, most of the studies conducted previously employed microarray analysis, which bares known limitations^37^.

Establishment and maintenance of latency in host after primary lytic infection is a hallmark of herpesvirus infection^38^. This study was designed to systematically investigate transcriptomic changes in the line 6_3_ and line 7_2_ chickens in response to a vv + MDV challenge at 10 and 21 dpi by RNA sequencing analysis, which include identification and characterization of differentially expressed genes (DEGs) between the two genetic lines with and without MDV infection during the latent and late cytolytic phases of MDV infection. Public web server tools for high-throughput genomics data analyses were employed to dissect the identified DEGs for functional interpretations and to select candidate DEGs functionally relevant to MD resistance, which was anticipated to provide some insights on the mechanisms of genetic resistance to MD.

## Results

### Global transcriptomic profiles of the lines 6_3_ and 7_2_

Total RNA was extracted from spleen samples of the inbred line 6_3_ and 7_2_ birds of both control and vv + MDV challenged groups at 10 and 21 dpi, which was subjected to RNA-seq analyses. A total of 198.28 million clean reads was generated with an average of 24.78 million reads per treatment group. Mapping rates of the clean reads to the chicken reference genome ranged from 86.4% to 88.9% (Supplementary Table S1). Expression level (reads) for each of the identified genes was normalized as the fragments per kilobase of transcript per million mapped reads (FPKM). A total of 11,426 and 11,227 genes for line 6_3_, 11,575 and 11,083 genes for line 7_2_ was identified in the control and MDV challenged groups, respectively, with FPKM values ≥1 at 10 dpi. A total of 11,343 and 10,922 genes for line 6_3_, 10,955 and 10,927 genes for line 7_2_ was identified in the control and MDV challenged groups, respectively, at 21 dpi. The total numbers of genes identified at 10 and 21 dpi were 12,132 and 11,793 with FPKM values ≥1, respectively, from both control and MDV challenged groups of the two lines.

**Figure 1:**
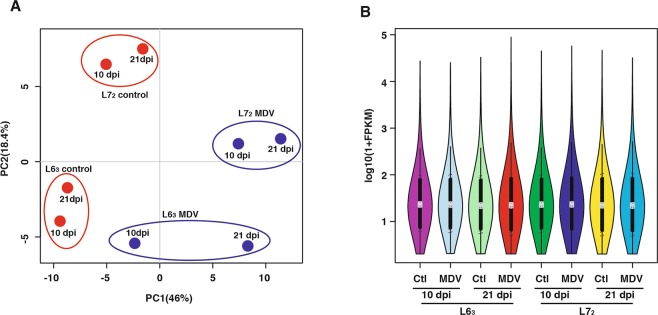
RNA-Seq overview. (**A**) A PCA score plot of 10,257 gene expression data, illustrating the clusters along the PC1 and PC2 coordinates by the treatment groups (MDV challenged vs. control) and genetic lines (6_3_ and 7_2_) of chickens; (**B**) A plot of FPKM distribution post normalization by chicken line and treatment groups, illustrating the overall uniformity of the expressional data across the treatment groups and the genetic lines of birds.

Overall gene expression levels were evidently clustered by treatment groups and by the genetic lines of chickens in a Principle Component Analysis (PCA)-space along the PC1 and PC2 coordinates, respectively, with one relatively notable deviation of the line 6_3_ MDV challenged group at 10 dpi, which was relatively less distanced from its control group and a bit more distanced from the group at 21 dpi (Fig. 1A). The average FPKM across all treatment groups was 87.0. Distributions of the FPKM were very comparable among the treatment groups (Fig. 1B), which suggested a general uniformity of the transcriptomes and basically free of global shift biases in transcriptomic levels among all the treatment groups. Therefore, it is highly likely that the differences detected in transcriptomic expression levels between the treatment groups and between the genetic lines were indeed due to the MDV challenge and the host genetics of different chicken lines, respectively.

### Differentially expressed genes in response to MDV challenge

**Figure 2:**
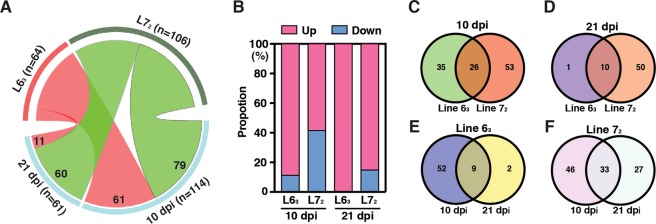
Graphical summaries of DEGs of line 6_3_ and 7_2_ birds in response to MDV challenge at 10 and 21 dpi by RNA-Seq. (**A**) The number of DEGs identified in each of the lines and in both lines at each of the time points. The length of the segments is proportional to the number of identified DEGs. (**B**) Illustrating the relative proportion of up- and down-regulated DEGs (red and blue portions of each bar, respectively) for each line at each time point. (**C**) A Venn diagram showing mutually exclusive DEGs and DEGs in common in response to MDV challenge at 10 dpi between the lines 6_3_ and 7_2_ groups. (**D**) A Venn showing mutually exclusive DEGs and DEGs in common in response to MDV challenge at 21 dpi between the lines. (**E**,**F**) Venn diagrams showing mutually exclusive and in common DEGs between the 10 and 21 dpi groups in response to MDV challenge for the line 6_3_ and line 7_2_ groups, respectively.

To improve the reliability and comparability of differential expression analysis, a total of 10,257 identified genes with FPKM values ≥1 in all treatment groups were included in this and subsequent analyses. At 10 dpi, 61 and 79 DEGs in response to MDV challenge were identified in the line 6_3_ and 7_2_ birds, respectively. Of which, 54 (88.52%) and 46 (58.22%) DEGs were significantly up-regulated in the line 6_3_ and 7_2_ birds, respectively (Fig. S1A,C). At 21 dpi, 11 and 60 DEGs in response to MDV challenge were identified in the line 6_3_ and 7_2_ birds, respectively. Interestingly, all the 11 DEGs of line 6_3_ were upregulated while 51 (85%) of the 60 DEGs in line 7_2_ were upregulated (Fig. 2A,B, and Fig. S1B,D). Detailed lists of all DEGs induced by MDV challenge in the two genetic lines at 10 and 21 dpi are given in Supplementary Tables S2–S5. As shown in the Venn diagrams (Fig. 2C,D), there were dozens of mutually exclusive DEGs between the lines 6_3_ and 7_2_ at 10 and 21 dpi except the line 6_3_ at 21 dpi with only a single mutually exclusive DEG, in addition to 26 and 10 DEGs identified in common between the lines of birds at 10 and 21 dpi, respectively. Within each of the lines between the two time points, 10 and 21 dpi, there were a few (2 in line 6_3_ at 21 dpi) up to dozens of DEGs identified mutually exclusive between the two time points, in addition to 9 and 33 DEGs in common between the two time points in line 6_3_ and line 7_2_, respectively (Fig. 2E,F).

### Deferentially expressed genes between the genetic lines with and without MDV challenge

**Figure 3:**
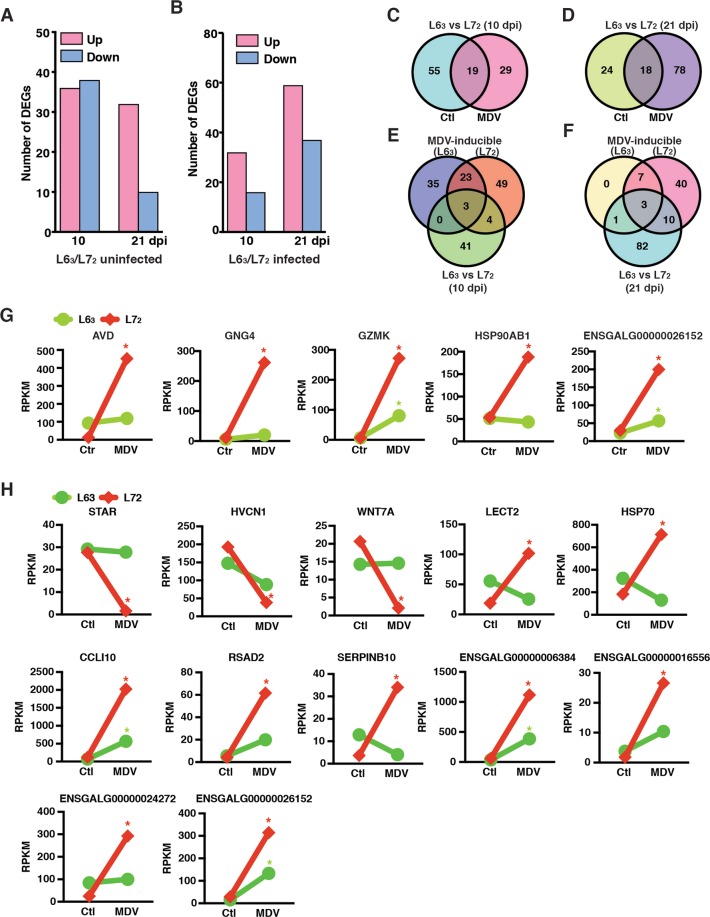
Depicting the numbers of DEGs identified between the resistant line 6_3_ and susceptible line 7_2_ birds without and with MDV challenge. (**A**) Number of DEGs significantly expressed higher (red bars) or lower (blue bars) in line 6_3_ birds in contrast to the line 7_2_ birds of the control groups at the ages matching the counterpart groups at 10 and 21 dpi. (**B**) Number of DEGs significantly expressed higher or lower in the line 6_3_ birds than in line 7_2_ birds in response to MDV challenge at 10 and 21 dpi. (**C**) A Venn diagram showing the number of DEGs in common (19) and mutually exclusive (55 and 29) between the line 6_3_ and 7_2_ control groups’ contrast and the MDV challenged groups’ contrast, respectively at 10 dpi. (**D**) A Venn diagram showing the number of DEGs in common (18) and mutually exclusive (24 and 78) between the line 6_3_ and 7_2_ control groups’ contrast and the MDV challenged groups’ contrast, respectively, at 21 dpi. (**E**) A Venn diagram showing the numbers of DEGs in common and mutually exclusive between the line 6_3_ challenged/control, line 7_2_ challenge/control, and the challenged groups of lines 6_3_ and 7_2_ at 10 dpi. (**F**) A Venn diagram showing the numbers of DEGs in common and mutually exclusive between the line 6_3_ challenged/control, line 7_2_ challenge/control, and the challenged groups of lines 6_3_ and 7_2_ at 21 dpi. (**G**) Depicting both the expression levels of 5 genes at 10 dpi and (H) 12 genes at 21 dpi without (control) and with MDV challenge between the line 6_3_ and line 7_2_ groups. A red star indicates a gene that was differentially expressed (FDR <0.05 and FC >2) in response to MDV challenge in the line 7_2_ birds, whereas a green star indicates that gene was differentially expressed (FDR <0.05 and FC >2) in response to MDV challenge in the line 6_3_ birds. Both groups of charts in G and H demonstrated that the difference in gene expression in the control groups did not alter the differential expression status of genes in response to MDV challenge, at least in this study.

In contrast to line 7_2_ control groups, 36 and 32 DEGs were significantly expressed at higher levels, 38 and 10 DEGs were expressed at lower levels in the line 6_3_ control groups at 10 and 21 dpi, respectively (Fig. 3A, Fig. S2A,B, and Tables S6, S7). Following MDV challenge, 32 and 59 DEGs were expressed at significantly higher levels, 16 and 37 DEGs were expressed significantly at lower levels in the line 6_3_ compared to the line 7_2_ at 10 and 21 dpi, respectively (Fig. 3B, Supplementary Fig. S2C,D, Supplementary Tables S8, S9). There were dozens of genes expressed differentially between the two lines regardless of MDV-challenge, and there were 19 and 18 genes differentially expressed in common between the two lines as well at 10 (Fig. 3C) and 21 dpi (Fig. 3D), respectively. In MDV-challenged birds, there were certain degrees of overlaps of genes differentially expressed between the two lines at 10 (Fig. 3E) and 21 dpi (Fig. 3F). A close examination of 5 and 12 genes at 10 and 21 dpi, respectively, between the lines 6_3_ and 7_2_ for both the control groups and the MDV challenged groups is depicted in Fig. 3G,H. The expression levels of those genes were significantly differentiated between the two lines, but none of the expression levels of those genes was between the control groups.

### ddPCR validation of the RNA-Seq data

To assess the validity of RNA-Seq data, a total of 12 pairs of primers (Table S10) was designed targeting 12 selected DEGs that were identified in a combination of 23 comparisons of this study by time point (dpi), MDV treatment and chicken lines. These primers were used in droplet digital^TM^ PCR (QX200TM ddPCR system; Bio-Rad Laboratories, Inc., Hercules, CA, USA) analysis for the validation. Three biological replicates were used for each treatment group. A scatterplot was generated by comparing the log_2_FC determined by RNA-Seq analysis and ddPCR analysis. The result showed there was a fairly high correlation between the two groups of log_2_FCs determined by the two methods (Fig. S3, R^2^ = 0.496, P value < 0.001). The ddPCR data validated the RNA-Seq data.

### ddPCR analysis of MDV microRNAs’ expressions associated with MDV latency

The expressions of two MDV microRNAs, miR-M3-3p and miR-M12-3p, in each of the total RNA samples extracted from MDV challenged birds and included in the current RNA_Seq analysis were quantitatively assessed by ddPCR to ensure that each of those total RNA sample of this study was from a challenged bird that had entered the latent phase. The miR-M3-3p and miR-M12-3p have been characterized with significantly higher expression in latent phases in contrast to that at the early cytolytic phase^39^. The ddPCR analysis was conducted with technical replicates using customer primers (Table S11). Splenic total RNA samples extracted from MDV challenged birds at 5 DPI were also used to contrast the difference of the microRNA expressions. The 5-DPI total RNA samples were aliquoted from a sister project conducted simultaneously along with this project using the same hatch of line 6_3_ and line 7_2_ birds under the same exact conditions. In a 20 μL reaction, an average of 7,020 ± 653.9 and 4,587 ± 610.0 miR-M3-3p copies at 10 DPI and 5,123 ± 866.6 and 3,750 ± 1,092.7 copies at 21 DPI was detected for lines 6_3_ and 7_2_, respectively; in contrast, an average of 422 ± 30.6 and 356 ± 35.9 copies was detected in the 5 DPI samples of line 6_3_ and 7_3_, respectively. The average of miR-M3-3p expression copies among all the 10 and 21 DPI individual total RNA samples ranged from 2,760 ± 180 to 8,120 ± 40 for line 6_3_, and 1,860 ± 20 to 6,470 ± 470 for line 7_2_.

The ddPCR quantification of the miR-M12-3p microRNA resulted in an average of 218 ± 17.7 and 197 ± 13.0 copies at 10 DPI, and 247 ± 18.3 and 195 ± 3.7 copies at 21 DPI for the line 6_3_ and 7_2_ samples, respectively. In contrast, an average of 63 ± 4.1 and 70 ± 8.4 copies at 5 DPI was detected for the lines 6_3_ and 7_2_, respectively. The average of miR-M12-3p copies expressed among all the individual birds at both 10 and 21 DPI ranged from 174 ± 12.0 to 300 ± 4.0, and 170 ± 6.0 to 216 ± 36.0 for lines 6_3_ and 7_2_, respectively.

The results of Analysis of Variance showed both MDV microRNAs’ expressions at 10 and 21 DPI significantly differed from those at 5 DPI (P < 0.01; leverage plots of the MDV microRNA expression data are given in Figs. S4 and S5 graphically illustrating the differences in expression at 5, 10, and 21 DPI). This in combination with the ranges of individual bird MDV microRNA expression evidently suggested that none of the MDV challenged birds under this study was remained at the early cytolytic phase.

### Functional analysis of the DEGs

To better understand the MDV-induced DEGs, functional enrichment analysis was conducted for four separate sets of the DEGs, which included two sets of MDV-induced DEGs in lines 6_3_ and 7_2_ at 10 dpi and two sets of MDV-induced DEGs in lines 6_3_ and 7_2_ at 21 dpi. At 10 dpi, the up-regulated genes in response to MDV challenge in line 6_3_ were primarily enriched in 13 GO terms, which are primarily associated with the immune system including immune effector process, immune system process, immune response, defense response, and regulation of immune response. The down-regulated genes in line 6_3_ at 10 dpi were associated with cell communication (*FAM132* *A*, *FN1* and *PROKR2*) and immune system process (*AQP3*). The up-regulated genes of line 7_2_ at 10 dpi were also significantly enriched in two GO terms of immune response and immune effector process. Some up-regulated DEGs of both lines 6_3_ and 7_2_ were also involved with the Influenza A pathway. In contrast, the line 7_2_ down-regulated genes were enriched in four GO terms and four KEGG pathways. Notably, these functional categories included several terms involved in fatty acid metabolism, such as fatty acid biosynthetic process, biosynthesis of unsaturated fatty acids and fatty acid metabolism (Table 1). Of the 11 significantly up-regulated genes in line 6_3_ at 21 dpi (Table S4), two were granzyme (*GZMA* and *GZMK*) genes, three were immune response genes (*MX*, OAS*A and *CCLI10*), one was an Interleukin Four Induced gene 1 (*IL4l1*) and one was avidin gene (*AVD*). For line 7_2_, the up-regulated DEGs were significantly enriched in GO terms primarily associated with immune response, including defense response, innate immune response, and response to interferon-gamma, in addition to influenza A pathway (Table 2).

**Table 1:** Significant enrichment of dysregulated gene sets in response to MDV challenge within each of the lines 6_3_ and 7_2_ at 10 dpi.

Line	Change	Accession	Description	Number of Genes	P value
Line 6_3_	Up-regulated	GO:0002252	Immune effector process	12	2.35E-06
		GO:0002376	Immune system process	19	5.91E-06
		GO:0006955	Immune response	14	9.52E-06
		GO:0043207	Response to external biotic stimulus	10	1.16E-03
		GO:0051707	Response to other organisms	10	1.16E-03
		GO:0006952	Defense response	12	1.31E-03
		GO:0009607	Response to biotic stimulus	10	1.80E-03
		GO:0009605	Response to external stimulus	15	3.08E-03
		GO:0051607	Defense response to virus	6	1.30E-02
		GO:0050776	Regulation of immune response	8	1.54E-02
		GO:0098542	Defense response to other organisms	7	2.21E-02
		GO:0050778	Positive regulation of immune response	7	4.26E-02
		GO:0009615	Response to virus	6	4.52E-02
		KEGG:05164	Influenza A	4	1.40E-02
Line 7_2_	Up-regulated	GO: 0006955	Immune response	13	1.42E-02
		GO:0002376	Immune system process	11	3.89E-04
		KEGG:05164	Influenza A	4	7.85E-03
	Down-regulated	GO:0006633	Fatty acid biosynthetic process	4	2.37E-02
		GO:0016053	Organic acid biosynthetic process	5	2.53E-02
		GO:0046394	Carboxylic acid biosynthetic process	5	2.53E-02
		GO:0044283	Small molecule biosynthetic process	6	2.84E-02
		KEGG:01040	Biosynthesis of unsaturated fatty acids	3	2.24E-04
		KEGG:01212	Fatty acid metabolism	3	2.19E-03
		KEGG:00982	Drug metabolism - cytochrome P450	2	3.77E-02
		KEGG:00980	Metabolism of xenobiotics by cytochrome P450	2	4.65E-02

**Table 2:** Significant enrichment of an up-regulated gene set in response to MDV challenge in the susceptible line 7_2_ birds at 21 dpi.

Accession	Description	Number of Genes	P value
GO:0006955	Immune response	13	1.02E-05
GO:0002376	Immune system process	17	2.88E-05
GO:0045087	Innate immune response	9	5.10E-05
GO:0002684	Positive regulation of immune system process	11	9.15E-05
GO:0006952	Defense response	12	2.10E-04
GO:0002682	Regulation of immune system process	12	8.13E-04
GO:0034341	Response to interferon-gamma	4	3.36E-03
GO:0045088	Regulation of innate immune response	6	4.52E-03
GO:0051704	Multi-organism process	12	6.74E-03
GO:0044764	Multi-organism cellular process	6	7.21E-03
GO:0060333	interferon-gamma-mediated signaling pathway	3	7.50E-03
GO:0009605	Response to external stimulus	13	1.86E-02
GO:0002252	Immune effector process	8	1.94E-02
GO:0006950	Response to stress	17	2.03E-02
GO:0043207	Response to external biotic stimulus	8	2.93E-02
GO:0051707	Response to other organisms	8	2.93E-02
GO:0050778	Positive regulation of immune response	7	3.30E-02
GO:0045089	Positive regulation of innate immune response	5	4.27E-02
GO:0009607	Response to biotic stimulus	8	4.37E-02
KEGG:05164	Influenza A	5	6.67E-04

Gene function analysis showed that DEGs that were relatively expressed at higher levels in the unchallenged line 6_3_ birds than those of the unchallenged line 7_2_ birds were significantly enriched in extracellular structure organization and extracellular matrix organization, as well as pathways of Focal adhesion and ECM-receptor interaction (Table 3). DEGs that were relatively expressed higher in the unchallenged line 7_2_ birds than those of the unchallenged line 6_3_ birds were mainly involved in metabolic pathways, such as pathways of Butanoate metabolism, Fatty acid metabolism, Biosynthesis of amino acids. Following infection at 10 dpi, six of the DEGs were immune genes (*GAL1*, *GAL2*, *GAL7*, *ABCB1*, *LEPR* and *SNED1*) and two were involved in NOD-like receptor signaling pathway (*K60* and *HSP90B1*), all were upregulated in expression in line 6_3_ birds. In contrast, four DEGs associated with response to stimulus (*HSP90AB1*, *GNG4*, *ANXA1*, *CD180*) were upregulated in expression in line 7_2_ birds. At 21 dpi, upregulated DEGs identified in line 6_3_ were significantly enriched in GO terms involved in system development and two pathways including Focal adhesion and ECM-receptor interaction, while upregulated DEGs identified in line 7_2_ were mainly associated in immunity, as indicated in enriched functional categories such as antigen processing and presentation, adaptive immune response, cell killing and KEGG pathway (Table 4).

**Table 3:** Significant enrichment of differentially expressed gene sets between line 6_3_ and 7_2_ control groups of birds.

DPI	Change	Accession	Description	Number of Genes	P value
10	Expressed significantly higher in line 6_3_	GO:0035092	Sperm chromatin condensation	2	4.95E-02
		KEGG:04512	ECM-receptor interaction	3	7.59E-03
		KEGG:04510	Focal adhesion	4	1.14E-02
	Expressed significantly higher in line 7_2_	GO:0044711	Single-organism biosynthetic process	10	2.51E-02
		KEGG:01100	Metabolic pathways	10	1.05E-03
		KEGG:01212	Fatty acid metabolism	3	3.11E-03
		KEGG:00072	Synthesis and degradation of ketone bodies	2	6.07E-03
		KEGG:01230	Biosynthesis of amino acids	3	6.96E-03
		KEGG:01040	Biosynthesis of unsaturated fatty acids	2	2.53E-02
		KEGG:00650	Butanoate metabolism	2	3.35E-02
		KEGG:00982	Drug metabolism - cytochrome P450	2	4.63E-02
21	Expressed significantly higher in line 6_3_	GO:0032501	Multicellular organismal process	30	3.13E-04
		GO:0044707	Single-multicellular organism process	28	6.94E-04
		GO:0007275	Multicellular organism development	23	1.06E-02
		GO:0044699	Single-organism process	46	1.31E-02
		GO:0048731	System development	21	3.62E-02
		KEGG:04512	ECM-receptor interaction	5	4.71E-05
		KEGG:04510	Focal adhesion	5	6.83E-03

**Table 4:** Significant enrichment of differentially expressed gene sets between line 6_3_ and line 7_2_ MDV challenged groups of birds.

DPI	Change	Accession	Description	Number of Genes	P value
21	Up-regulated in line 6_3_	GO:0032501	Multicellular organismal process	30	3.13E-04
		GO:0044707	Single-multicellular organism process	28	6.94E-04
		GO:0007275	Multicellular organism development	23	1.06E-02
		GO:0044699	Single-organism process	46	1.31E-02
		GO:0048731	System development	21	3.62E-02
		KEGG:04512	ECM-receptor interaction	5	4.71E-05
		KEGG:04510	Focal adhesion	5	6.83E-03
	Up-regulated in line 7_2_	GO:0002711	Positive regulation of T cell mediated immunity	6	3.13E-07
		GO:0002709	Regulation of T cell mediated immunity	6	9.15E-07
		GO:0002456	T cell mediated immunity	6	2.55E-06
		GO:0002708	Positive regulation of lymphocyte mediated immunity	6	5.49E-06
		GO:0002824	Positive regulation of adaptive immune response based on somatic recombination of immune receptors built from immunoglobulin superfamily domains	6	6.08E-06
		GO:0002821	Positive regulation of adaptive immune response	6	6.72E-06
		GO:0042026	Protein refolding	4	6.88E-06
		GO:0006457	Protein folding	7	1.08E-05
		GO:0002705	Positive regulation of leukocyte mediated immunity	6	1.08E-05
		GO:0002706	Regulation of lymphocyte mediated immunity	6	2.16E-05
		GO:0002822	Regulation of adaptive immune response based on somatic recombination of immune receptors built from immunoglobulin superfamily domains	6	3.45E-05
		GO:0002819	Regulation of adaptive immune response	6	4.61E-05
		GO:0002703	Regulation of leukocyte mediated immunity	6	8.47E-05
		GO:0002699	Positive regulation of immune effector process	6	1.74E-04
		GO:0002449	Lymphocyte mediated immunity	6	2.06E-04
		GO:0002460	Adaptive immune response based on somatic recombination of immune receptors built from immunoglobulin superfamily domains	6	3.65E-04
		GO:0001916	Positive regulation of T cell mediated cytotoxicity	4	5.60E-04
		GO:0001914	Regulation of T cell mediated cytotoxicity	4	7.55E-04
		GO:0002443	Leukocyte mediated immunity	6	8.71E-04
		GO:0001913	T cell mediated cytotoxicity	4	9.98E-04
		GO:0002250	Adaptive immune response	6	1.03E-03
		GO:0001912	Positive regulation of leukocyte mediated cytotoxicity	4	2.58E-03
		GO:0031343	Positive regulation of cell killing	4	3.49E-03
		GO:0001910	Regulation of leukocyte mediated cytotoxicity	4	3.49E-03
		GO:0031341	Regulation of cell killing	4	4.62E-03
		GO:0001909	Leukocyte mediated cytotoxicity	4	7.68E-03
		GO:0001906	Cell killing	4	1.20E-02
		GO:0002697	Regulation of immune effector process	6	1.74E-02
		GO:0002474	Antigen processing and presentation of peptide antigen via MHC class I	3	4.69E-02
		GO:0050778	Positive regulation of immune response	6	4.84E-02
		GO:0035745	T-helper 2 cell cytokine production	2	4.86E-02
		GO:2000551	Regulation of T-helper 2 cell cytokine production	2	4.86E-02
		GO:2000553	Positive regulation of T-helper 2 cell cytokine production	2	4.86E-02
		KEGG:04141	Protein processing in endoplasmic reticulum	4	5.46E-03

### Key genes for MD resistance

**Figure 4:**
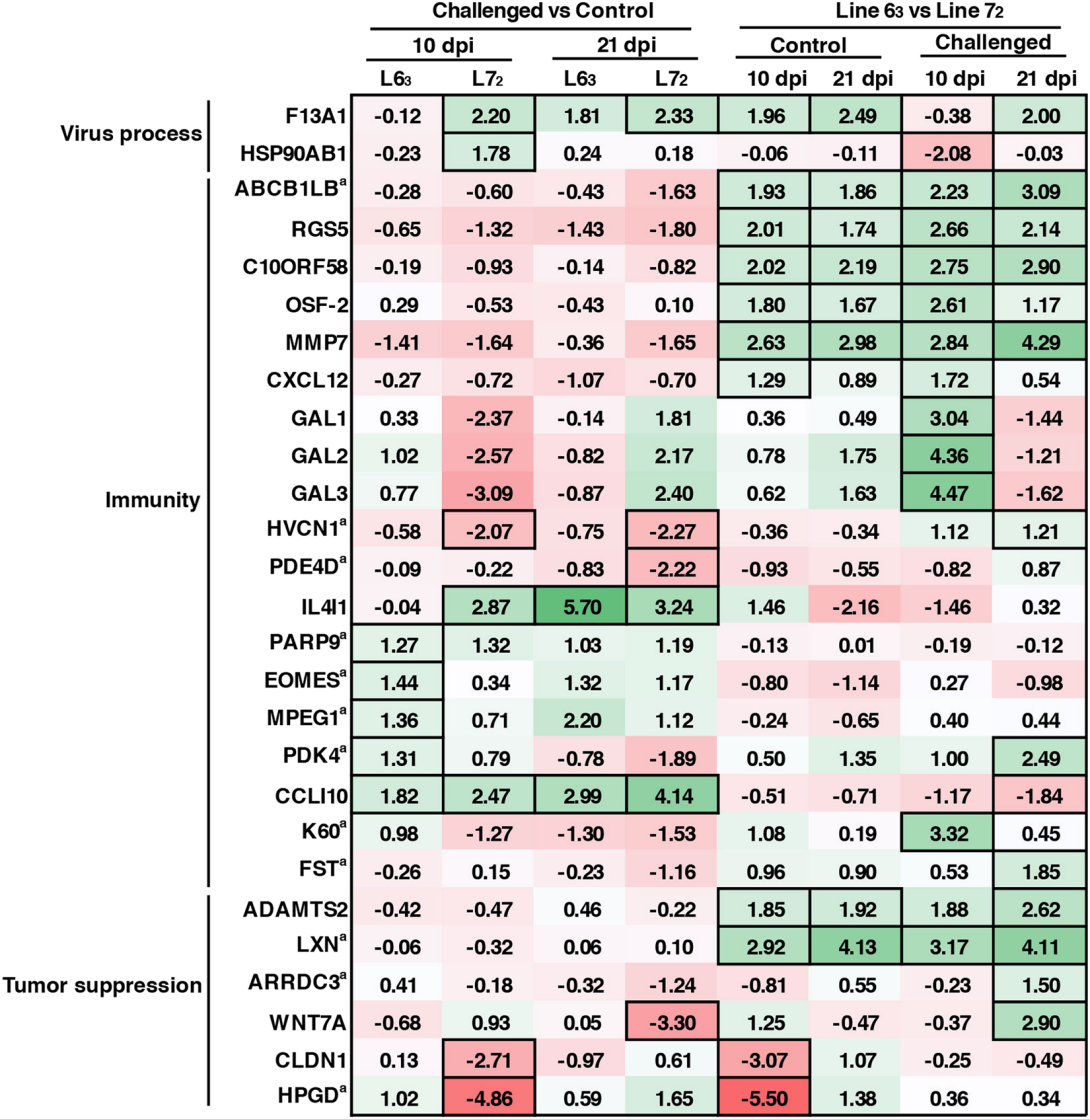
Heat map showing fold changes for 27 promising candidate DEGs under each of the pairwise comparisons. The statistically significant fold change (FC) for those genes are individually boxed (FDR <0.05 and FC >2). Those genes with a superscript a, “^a^”, were reportedly located within MD-QTL regions.

To further identify key genes that may confer genetic resistance to MD from all the unique DEGs, we focused on the followings: (1) DEGs located within reported QTL regions of MD resistance (http://www.animalgenome.org/cgi-bin/QTLdb/GG/index); (2) DEGs with large fold change (FC >2) in one line but not in the other (FC < 0.5); (3) DEGs differentially expressed between the two lines regardless of MDV challenge; (4) MDV induced DEGs with differential expression between infected line 6_3_ and line 7_2_ birds (Fig. 3E–H). These comparisons resulted in a total of 104 unique DEGs. Each of these DEGs was subjected to a separate Gene Ontology (GO) analysis and a comprehensive literature review. Jointly considering the expression pattern of each gene in different chicken lines and the potential functionality, a short list of 27 top DEGs was selected as the likely candidate genes identified from this study, which may potentially play key roles in conferring genetic resistance to MD (Fig. 4). These genes were broadly grouped into three functional groups, including virus process, immunity and tumor suppression.

## Discussion

Several studies have documented the global gene expression responses to MDV infection in chicken^3,12,28–36^. These analyses, while providing valuable and important mechanistic clues to host responses and virus-host interactions, are of limited scope due to coverage of gene arrays used and lack information of comparisons between chickens exhibiting different resistance to MD. By this study, we further expanded the current knowledge with regard to host transcriptomic response upon MDV challenge through comparing the whole transcriptome changes between a highly inbred MD-resistant and -susceptible lines of chicken using RNA-Seq technology. This study was primarily focused on the latent stages post MDV inoculation. Our results revealed quite different transcriptome patterns, which may provide new insights potentially conferring genetic resistance to MD.

Close examinations of the transcriptomic changes in response to MDV challenge showed that many genes associated with immunity and anti-tumor functions were activated in both lines of chickens. For example, two granzyme genes, *GZMA* and *GZMK*, that are associated with apoptosis, and the *MX1* gene that is a well-known IFNs-induced gene^40^, were up-regulated in both lines at 10 and 21 dpi, suggesting on going interactions that took place between host and the MDV pathogen. In contrast, more different features in response to MDV infection between the two inbred lines were observed. More DEGs were identified in the MD susceptible line 7_2_ than those in the resistant line 6_3_ at both 10 and 21 dpi (Fig. 2A), indicating that MDV induced much stronger response at the transcriptional level in line 7_2_ than in line 6_3_ birds. However, it is interesting to note that the proportion of up-regulated DEGs was much higher in line 6_3_ than that in line 7_2_ (Fig. 2B), which suggested that insufficient immune response was probably activated against tumorigenesis regardless more immune-related gene expressions were altered in response to MDV challenge. Specifically, in MD resistant line 6_3_, a notable immune response was observed at 10 dpi, which was characterized by the overrepresentation of DEGs enriched in many immune response-related GO terms, such as immune response, defense response to virus, positive regulation of immune response, and so on. While at 21 dpi, only 11 genes were identified to exhibit a statistically significant change in expression, indicative of a minimal change compared to normal tissues. In susceptible line 7_2_, a totally different transcription pattern was revealed across the two-time points. At 10 dpi, 33 out of 79 DEGs (41.77%) were down-regulated in expression (Fig. 2B, Fig. S2C). These down-regulated genes were significantly enriched in multiple GO terms and pathways, including several terms associated with fatty acid metabolism, such as fatty acid biosynthetic process, pathways of fatty acid metabolism, and biosynthesis of unsaturated fatty acids (Table 2). Genes involved in these terms included *SCD*, *FADS1*, *FADS2* and *HPGDS*. Previous studies showed that Hepatitis C virus (HCV) could induce imbalance in lipid homeostasis in host cells^41,42^. However, it seemed that inhibition of fatty acid biosynthesis can suppress virus replication^43,44^ and control cancer cell proliferation^45^. Therefore, whether the down-regulation of these genes in susceptible line 7_2_ playing a role in establishment of latency or representing a host response that may contribute to repressing the activities of MDV infection needs to be further investigated. At 21 dpi, 60 DEGs were identified being differentially expressed compared to the control birds and 51 of those were up-regulated. Gene function analysis showed that the up-regulated genes were significantly enriched in immune response-related terms such as innate immune response, interferon-gamma-mediated signaling pathway, and so on. In particular, the *IRF1* (Interferon regulatory factor-1) gene expression was upregulated by 4.2 folds (from an FPKM of 1278.1 to 5415.7). *IRF1* is a member of the *IRF* gene family of transcription factors that binds to the virus-inducible *cis*-elements of *IFN*-*α* and *IFN*-*β* gene promoter as well as to the interferon response sequence of IFN-inducible gene promoters^46^. Accordingly, the 51 identified up-regulated DEGs were significantly enriched in IRF-1 (*P* value = 5.66E-5) by a motif enrichment analysis using the g:Profilier (http://biit.cs.ut.ee/gprofiler/). A total of 15 up-regulated genes containing potential IRF-1 binding site was revealed, including *IFIH1*, *BCL2L14*, *GZMK*, *ISG12*(*2*), *SERPINB10*, *IFI27L2*, *AVD*, *TAP1*, *CCL19*, *CMPK2*, *ENSGALG00000026152*, *ENSGALG00000001629*, *ENSGALG00000006384*, *ENSGALG00000013057* and *ENSGALG00000019141*. These gene functions implied strong defense responses might have been activated in susceptible line 7_2_ at 21 dpi, which is in good agreement with earlier observations that MDV were reactivated in susceptible line of birds at late latency stage^1^. Furthermore, we observed some genes showed extreme difference in response to MDV challenge between the two genetic lines of birds at each time points (Fig. S2C,D). These genes might also play key roles modulating genetic resistance to MD, therefore, follow-up investigations are warranted. One of those DEGs is *IL4I1*. Reportedly it is an inhibitor of the CD8+ antitumor T-cell response and may facilitate tumor growth. Our data showed that the expression of *IL4I1* was significantly increased in the susceptible line 7_2_ birds at 10 dpi, while it remained unchanged in the resistant line 6_3_ birds at the same time point, which indicated that genes like *IL4I1* may contribute to immunosuppression and facilitate tumorigenesis through inhibiting the CD8+ antitumor T-cell response in MD susceptible chickens. If so, it is then in good agreement with the functional findings of this gene in mice^47^.

Differences in gene expression levels were also observed between the two lines at both time points for birds without and with MDV challenge. Notably, genes associated with Focal adhesion and ECM-receptor interaction were observed with higher expression in the resistant line 6_3_ control birds (Table 3). Interestingly, following MDV challenge, genes involved with these two pathways exhibited consistently elevated expression in the resistant line 6_3_ birds than that in the susceptible line 7_2_ birds at 21 dpi (Table 3), indicative of potential roles of these two pathways in MD resistance. The Focal adhesion and ECM-receptor interaction pathways have a profound influence on major cell programs including growth, differentiation, migration, and survival^48^, which suggests differences in functional properties of cells between the resistant and the susceptible lines of chicken. Furthermore, earlier studies have documented that these two pathways play a major role in immunity^49,50^. More interestingly, these pathways are also reportedly linked to tumor progression^51,52^. Therefore, the higher expression of these genes involved in Focal adhesion and ECM-receptor interaction may be contributing to tumor formation and development. Notably, the majority of the significantly enriched functional categories for DEGs with higher levels of transcriptional expression were observed in the susceptible line 7_2_ birds other than in the resistant line 6_3_ birds in response to MDV challenge, which are related to immune response, including positive regulation of T cell mediated immunity, adaptive immune response, and positive regulation of cell killing. This, again, implicated the strong reactivation of the susceptible line 7_2_ birds to MDV infection at these points of time.

Merging the DEGs identified between the infected and uninfected birds as well as between the two lines resulted to a total of 284 unique genes. Among them, 189 DEGs (66.6%) have been previously reported in studies of host response to MDV challenge^3,12,28–36^, including genes associated with chemokine (*CCL1*, *CXCL13L2*, *CXCL12* and *CCL19*), cytokine (*IL21R*, *IL2RA*, *LEPR*, *IL12RB2*, *FLT3*), innate immune response (*IRF1*, *STAT1*, *CD36*, *IFIH1*, *SLC11A1*, *IGJ*, *PAPR9*, *GCH1*, *LAG3*, *RSAD2* and *HPX*), and adaptive immune response (*B2M*, *P2RX7*, *SLC11A1*, *ANXA1*, *RSAD2*, *HPX*, and *BFIV21*). This overlap confirms the roles of these candidate genes that may play important roles in response to MDV challenge. On the other hand, due to other differences including genetic background of chickens, MD virus strains, tissue samples and sampling time, novel DEGs were identified in this study, which expanded the candidate gene pool conferring genetic resistance to MD. Jointly considering the gene expression pattern in two lines, gene function, and comparison with QTL regions, 27 promising top candidate genes were proposed based on this study, which may highly likely play key roles in MD including conferring genetic resistance to MD (Fig. 4). These genes showed large differences either between resistant and susceptible lines, or in response to MDV challenge within each of the genetic lines, or both, or are located at reported MD-related QTL regions. Functional analysis classified these genes into three broadly functional categories, including viral process, immunity, and tumor development.

Earlier studies have shown that viral load was different between the line 6_3_ and line 7_2_ at latency phase of infection^25,26^. Consistent with these observations, we found two genes, *F13A1* and *HSP90AB1*, with known functions in promoting virus replication^53,54^, that were significantly induced in expression in response to MDV challenge in the susceptible line 7_2_ birds, but with little change in the resistant line 6_3_ birds at 10 dpi. Presumably, these two genes might be hijacked by MDV to facilitate viral replication in the susceptible line 7_2_ birds. Additionally, there were 19 genes that may potentially play roles in immunity. Among them, *ABCB1LB*, *RGS5*, *C10ORF58*, *OSF*-*2*, *MMP7* and *CXCL12* were observed with higher expression levels in the resistant line 6_3_ birds regardless of MDV challenge treatment. Our results also revealed that some immune response-related genes, including *HVCN1* and *PDE4D*, were down-regulated in the susceptible line 7_2_ birds in response to MDV challenge. Together with the identification of up-regulation of *IL4I1* and down-regulation of defensing genes, *GAL1*, *GAL2* and *GAL3*, in the susceptible line 7_2_, it is postulated here that the susceptible line 7_2_ birds may suffer from immunosuppression at latent stage of MDV infection, which is in good agreement with a previous report^29^. Other promising candidates involved in immunity were uniquely induced by MDV in the resistant line 6_3_ (*PARP9*, *EOMES*, *MPEG1*, *PDK4*, *CCLI10*, *K60* and *FST*) compared to the susceptible line 7_2_. Presumably, these genes may belong to the group that contribute to genetic resistance to MD. MD is a virus-induced tumorous disease of chicken and has been proposed to be an invaluable model for investigation of virus-induced cancers^7,55,56^. Our results showed that six putative tumor suppressor genes, including *ADAMTS2*^57^, *LXN*^58^, *ARRDC3*^59^, *WNT7A*^60^, *CLDN1*^61^ and *HPGD*^62–64^, exhibited higher expression levels in the resistant line 6_3_ than in the susceptible line 7_2_ birds, which should confer genetic resistance to MD. The findings from this study underscored the value of MD study serving as a model for better understanding tumorigenesis and raised the possibility that more novel genes involved in tumorigenesis and development need to be further explored.

## Conclusion

In summary, we have carried out a comprehensive gene expression study using RNA-Seq analysis and identified hundreds of differentially expressed genes in response to a vv + MDV challenge in two highly inbred lines of chickens at 10 and 21 days post MDV infection. It is well-documented that one of the lines, the line 6_3_, is relatively resistant to MD, while the other, the line 7_2_, is highly susceptible to MD. We identified a total of 284 unique coding genes that likely affect the resistance to MD, which provided a sizable and valuable addition to the current candidate gene pool that is reportedly involved with genetic resistance to MD. We showed that the response pattern of gene expression at 21 dpi supported a reactivation of MDV in the susceptible line. We further proposed 27 promising candidate genes that may play key roles conferring genetic resistance to MD. Notably, most of these promising candidate genes identified in this study are reportedly associated with immunity and tumor suppression, which may have an important implication on virus-induced tumorigenesis in general and highlighted the value of MD model for tumor virus-induced cancer study. Further directions should include work on the detailed function of these candidate genes and regulatory control of the expression of those genes upon explosion to tumor viruses like MDV.

## Materials and Methods

### Experimental design

One-day old chickens from two highly inbred lines were sampled for an MDV challenge trial in this study. One genetic line is known as line 6_3_ and the other, as line 7_2_. The two genetic lines were developed and have been maintained at the USDA, Agricultural Research Service, Avian Disease and Oncology Laboratory (ADOL) in East Lansing, Michigan. The lines 6_3_ and 7_2_ share a common major histocompatibility complex (B*2) haplotype but are resistant and highly susceptible to MD, respectively^15^. On the day of hatch, chickens from each line were randomly divided into MDV challenge group and control group. Each of the chicks in the MDV challenge groups of both lines was inoculated intraabdominally with 500 plaque-forming units of 648A passage 10 MDV at day 5 post hatch. No inoculation was implemented in the control groups. Three chickens from each group were randomly euthanized at 10 (latency period) and 21 dpi (reactivation period), respectively. Spleen samples were individually collected, immediately placed into RNAlater solution (Qiagen, Valencia, CA, USA), and stored at −20 °C until total RNA extraction.

All chickens used in this study were housed in a BSL-2 experimental facility during the trial. Feed and water were supplied *ad libitum*. The chickens were observed daily throughout the entire duration of the experiment. The animal challenge experiment was approved by the USDA, Avian Disease and Oncology Laboratory Institutional Animal Care and Use Committee (IACUC). The IACUC guidelines established and approved by the ADOL IACUC (April 2005) and the Guide for the care and use of Laboratory Animals by Institute for Laboratory Animal Research (2011) were closely followed throughout the experiment.

### RNA sequencing

Total RNA was extracted using TRIzol reagent (Invitrogen, Carlsbad, CA, USA) following the manufacturer’s instructions. RNA concentration and quality were assessed using an Agilent 2100 Bioanalyzer (Agilent Technologies, Santa Clara, CA, USA). Equal amount of RNA samples from three biological replicates within each line each treatment group were pooled in preparation to construct standard cDNA libraries using Illumina TruSeq kits and reagents following the manufacturer’s protocol for deep sequencing. The libraries were sequenced on an Illumina HiSeq2000 sequencer for single end 50 base sequencing run. The post sequencing processes, including image analysis, base calling, and Q-Score calculation, were carried out using Real Time Analysis (v1.13.48); read demultiplexing and conversion to final FASTQ files, using CASAVA (v1.8.2) software tools (Illumina Inc., San Diego, CA, USA). The library preparation, RNA sequence read extraction, and preliminary read quality control were performed at the Research Technology Support Facility, Michigan State University.

### Mapping and gene expression quantitation

Sequence adaptors were removed in the first quality control process using Trimmomatic (version 0.32) software^65^ to obtain the pass-filter (PF) reads. Low quality bases were further trimmed from the PF reads using custom Python scripts eliminating the first 15 nucleotides. Sickle (v1.33)^66^ was used with a sliding window average score of 30 in removing reads with “N”s, and minimum read length of 30 bps, and ended with clean reads. The clean reads were then used to map to the chicken genome reference (galGal4) using TopHat2 (v2.0.12)^67^ and Bowtie2 (v2.2.3)^68^ with default parameters. Transcript abundance and differential expression of genes were estimated with Cufflink (v2.2.1)^69^. FPKM values were obtained to quantify relative expression of transcripts.

### Analyses of DEGs between treatment and chicken line groups

The number of reads per gene for each sample were counted using HTSeq^70^. In each of the pairwise comparisons (between infected birds and control in each line, and between the two lines with and without MDV infection), DEGs were identified by using the DESeq R package (2.1.0) and selected using a filter criterion of FDR <0.05 and FC >2. To better understand the functional involvements of these DEGs, g:Proflier (http://biit.cs.ut.ee/gprofiler/index.cgi)^71^ were used for the gene annotation, GO term and pathway enrichment analyses.

### Droplet Digital^TM^ PCR validation of gene expression

To validate sequencing data, three genes from each of the treatment groups were selected and re-evaluated on a Droplet DigitalTM PCR (QX200TM ddPCR system; Bio-Rad Laboratories, Inc., Hercules, CA, USA). The primers for ddPCR were designed with Primer3Plus (http://www.bioinformatics.nl/cgi-bin/primer3plus/primer3plus.cgi/) and are listed in Table S10. A total of seven genes was selected. The cDNA samples used in ddPCR validation were reversely transcribed from individual RNA samples using the iScript^TM^ RT Supermix Kit (Cat No. 170–8841) and following the manufacturer’s instructions (Rio-Rad). Those total RNA samples were the same samples used in cDNA library preparation for the RNA-Seq analysis of this study. A ddPCR reaction mixture of 25 μL in final volume was initially prepared per gene per biological sample including 2 μL of cDNA, 12.5 μL of EvaGreen Supermix (Cat No. 1864034), 0.5 μL of each of the forward and reverse primers (200 nM; synthesized by Eurofins Genomics, Huntsville, AL), and 9.5 μL of nuclease-free water. Of which, 20 μL were loaded into one of 8 sample channels of a DG8^TM^ cartridge (Cat No. 1864008, Bio-Rad). Each oil well was loaded with 70 μL of droplet generating oil (Cat No. 1864006, Bio-Rad). The loaded DG8^TM^ cartridges were placed on a QX200^TM^ droplet generator (Bio-Rad) to generate the digital droplets. Forty μL of the generated droplet emulsion for each sample were transferred to a well in a 96-well PCR plate followed by polymerase chain reaction with EvaGreen on a C1000^TM^ Thermal Cycler (Bio-Rad). The cycling conditions were 95 °C for 5 min, followed by 40 cycles of 95 °C for 15 s, 58 °C for 60 s, and a final extension step of 98 °C for 10 min. The droplets post PCR were read well by well on a QX200^TM^ droplet reader (Bio-Rad). PCR-positive and PCR-negative droplets in each of the wells were counted and analyzed with the QuantaSoft^TM^ Software (Version 1.7, Bio-Rad).

Following similar procedures, two MDV microRNA expression levels in each of the total RNA samples included in this study were determined by ddPCR using customer primers (Table S11) to ensure that all the MDV challenged birds included in the samplings had entered the latent phase.

## Supplementary information

Supplimentary File 1

Supplementary File 2
