# Supplementary material for: RNA Sequencing revealed differentially expressed genes functionally associated with immunity and tumor suppression during latent phase infection of a vv + MDV in chickens
Source: Sci Rep. 2019 Oct 2;9:14182. doi: 10.1038/s41598-019-50561-x (PMC6775254; doi:10.1038/s41598-019-50561-x)
Supplement: Supplementary file 1 — Supplimentary File 1 [file 41598_2019_50561_MOESM1_ESM.pdf]

Supplementary files

RNA Sequencing revealed differentially expressed genes functionally associated with immunity and tumor suppression during latent phase infection of a vv+MDV in chickens

Kunzhe Dong[1,2], Shuang Chang[3], Qingmei Xie[4], Peng Zhao[3], and Huanmin Zhang[1*]

[1]USDA, Agricultural Research Service, Avian Disease and Oncology Laboratory, East Lansing, MI 48823, U.S.A.

[2]ORISE Fellow, USDA, Agriculture Research Service, Avian Disease and Oncology Laboratory, East Lansing, MI 48823, U.S.A.

[3]College of Veterinary Medicine, Shandong Agricultural University, Tai'an, Shandong 271018, China

[4]College of Animal Science, South China Agricultural University, Guangzhou 510642, China

To whom all correspondence should be addressed: Huanmin.Zhang@ars.usda.gov

Phone: 01-(517) 337-6835

Fax: 01-(517) 337-6776

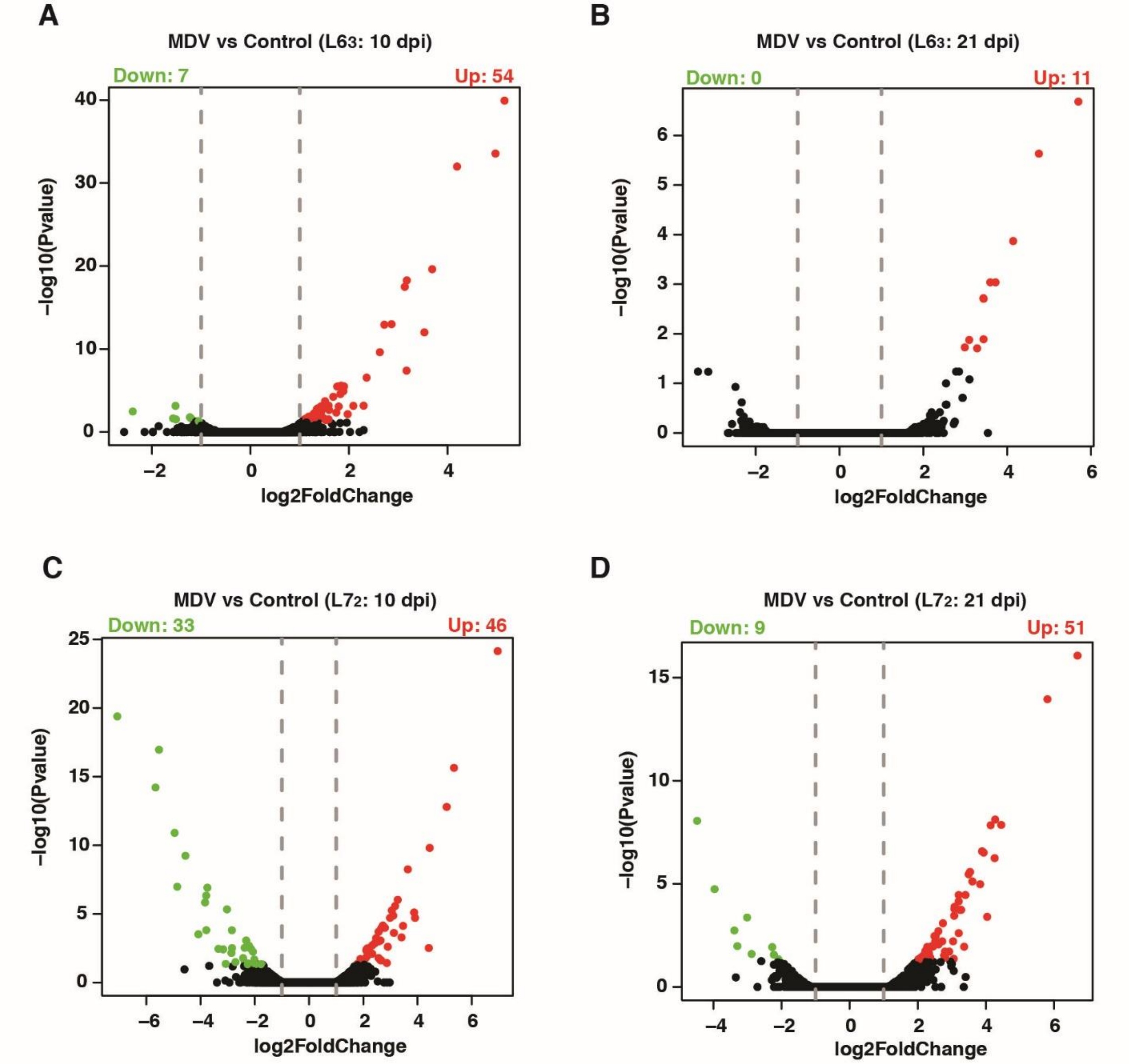

Figure S1. Differentially expressed genes in response to MDV challenge identified in line 6_3 and line 7_2 at 10 and 21 dpi. Volcano plots showing the differentially expressed genes in response to MDV challenge in line 6_3 at 10 dpi (A) and 21 dpi (B); in line 7_2 at 10 dpi (C) and 21 dpi (D).

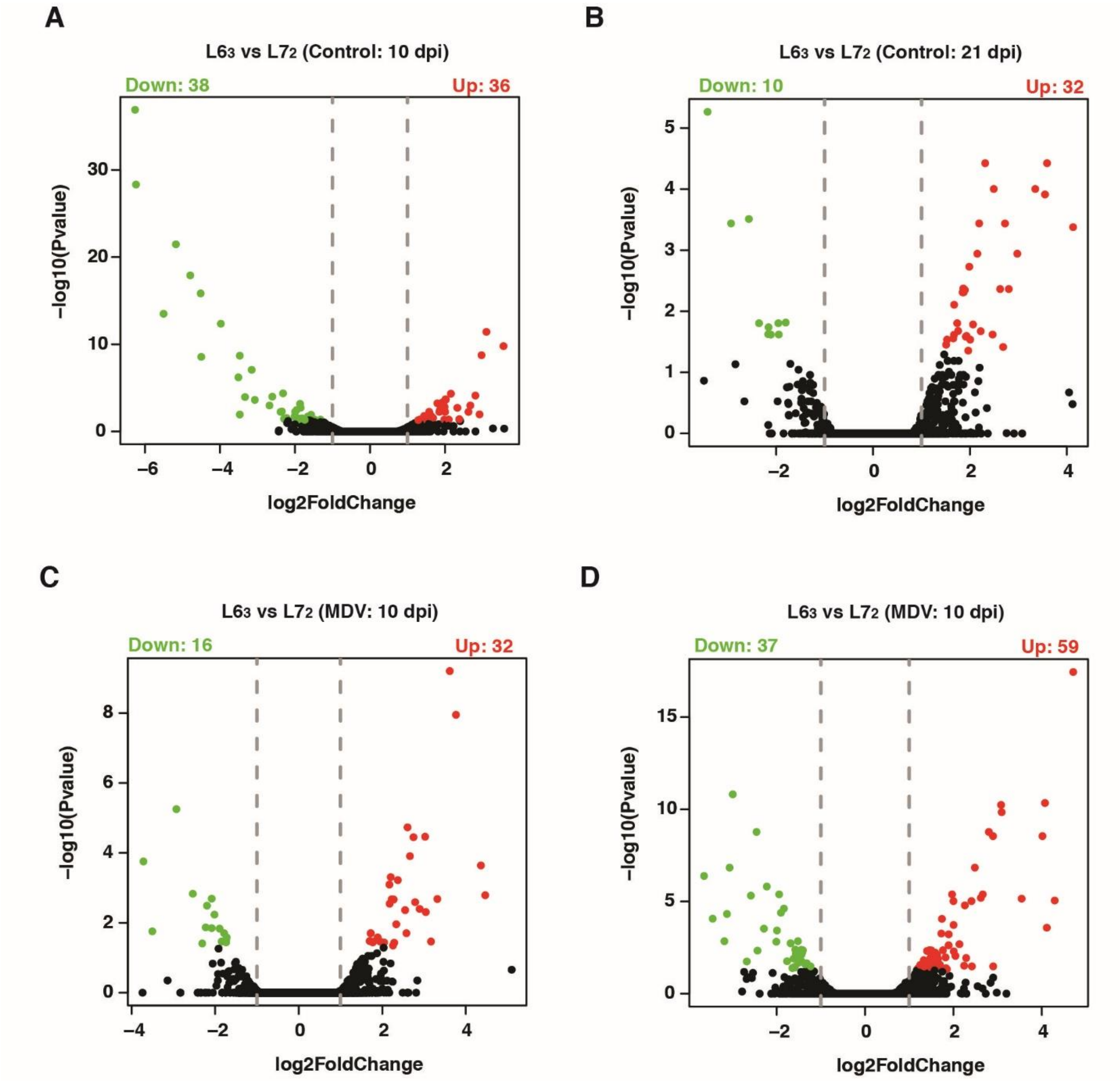

Figure S2. Differentially expressed genes between non-challenged and challenged line 6_3 and line 7_2 at 10 and 21 dpi. Volcano plots showing the differentially expressed genes between non-challenged line 6_3 and line 7_2 birds at 10 dpi (A) and 21 dpi (B); and between challenged line 6_3 and line 7_2 at 10 dpi (C) and 21 dpi (D).

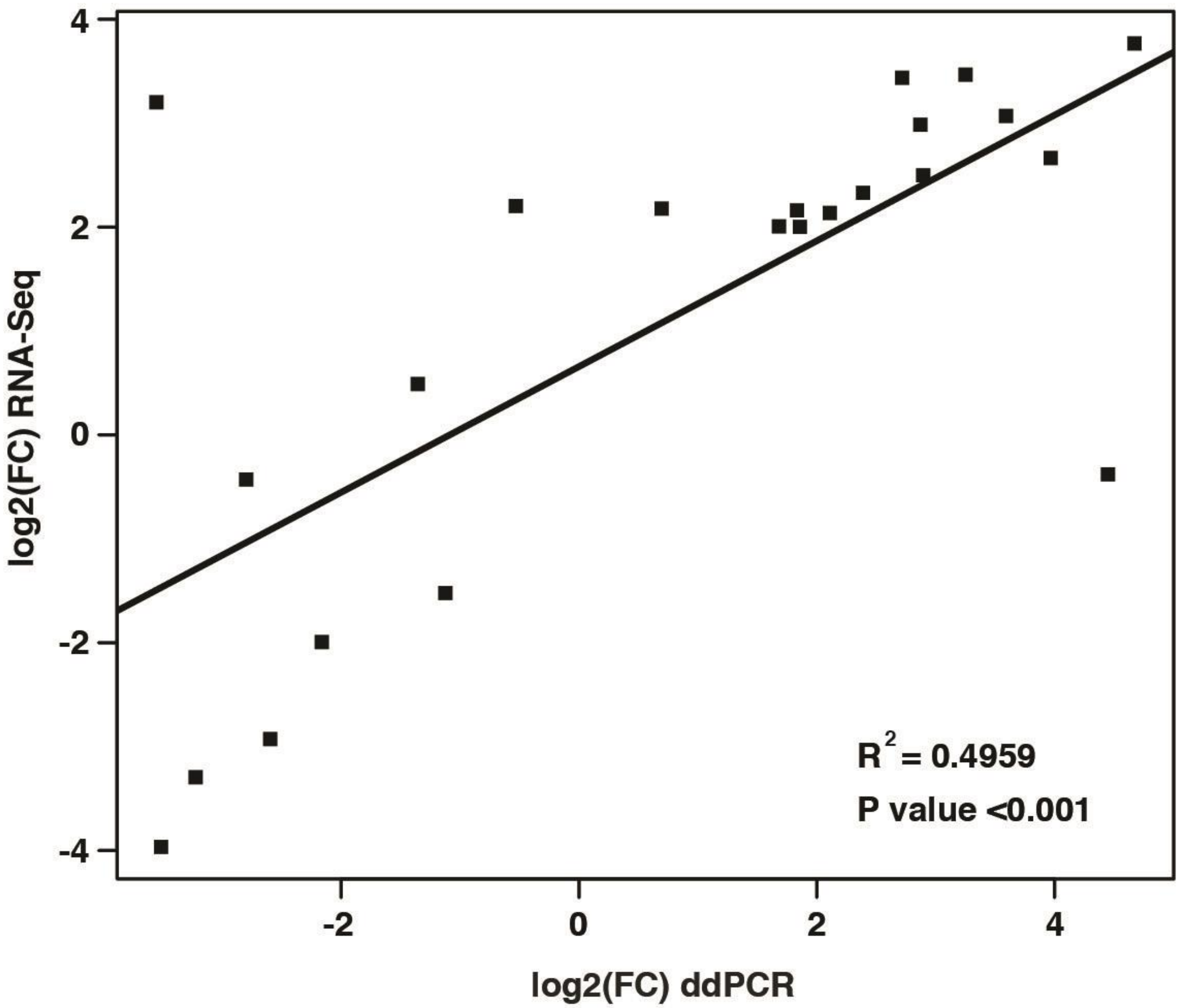

Figure S3. A plot showing correlation between the expression levels of a selected subgroup of genes identified by RNA-Seq and re-tested with ddPCR. The overall correlation coefficient for the selected subgroup of genes in test was r = 0.7, which validated the expression data acquired by RNA-Seq with a reasonable confidence.

Line 6_3 total RNA samples

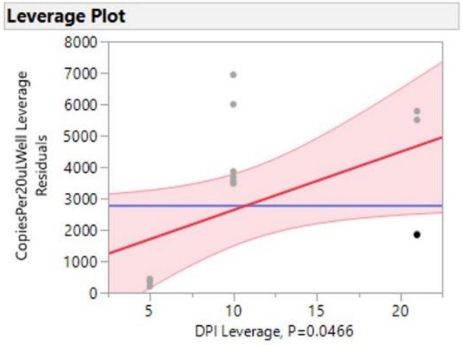

Line 7_2 total RNA samples

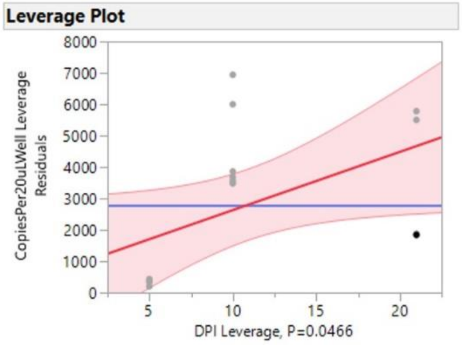

Figure S4. MDV miR-M3-3p microRNA expression leverage plots based on ddPCR absolute quantifications of the microRNA copies per 20-µl reaction at 5, 10 and 21 days post MDV challenge for each individual sample with technical replicates (the 5 dpi total RNA samples were borrowed from a sister project, which was conducted simultaneously under the exact same conditions using the same batch of line 6_3 and 7_2 chicks). It is clearly shown that the miR-M3-3p microRNA expressions of all birds at 10 and 21 dpi differed from the expressions at 5 dpi in both line 6_3 and line 7_2 birds.

Line 6_3 total RNA samples **Line 7_2 total RNA samples**

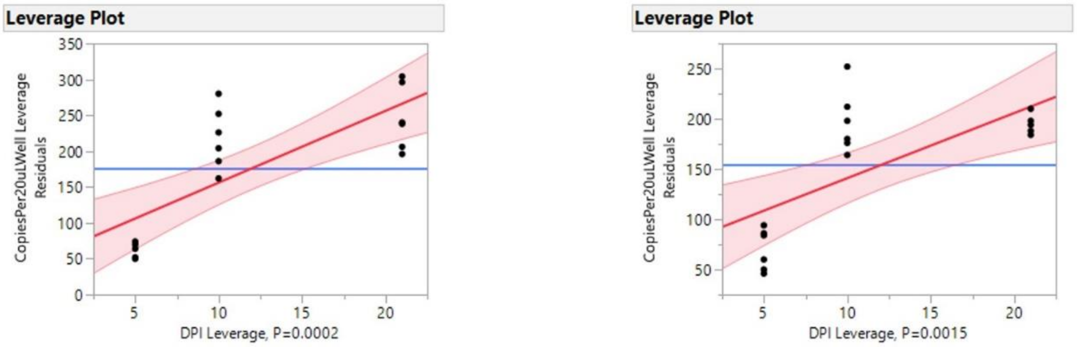

Figure S5. MDV miR-M12-3p microRNA expression leverage plots based on ddPCR absolute quantifications of the microRNA copies per 20-µl reaction at 5, 10 and 21 days post MDV challenge for each individual sample with technical replicates. It is clearly shown that the miR-M12-3p microRNA expression of all birds at 10 and 21 dpi also differed from the expressions at 5 dpi in both line 6_3 and line 7_2 birds.

Supplementary Tables

Table S1. Numbers of reads aligned and mapped

Treatment group	Past filter reads	Clean reads	Mapped reads	Mapped rate (%)
$Line6_3$ 10 dpi Control	28,932,041	23,002,189	20,042,414	87.1
$Line6_3$ 10 dpi Challenged	27,792,916	22,147,027	19,434,748	87.8
$Line6_3$ 21 dpi Control	34,265,288	28,399,403	24,776,271	87.2
$Line6_3$ 21 dpi Challenged	26,638,242	21,678,806	18,826,655	86.8
$Line7_2$ 10 dpi Control	34,642,123	27,243,083	23,876,203	87.6
$Line7_2$ 10 dpi Challenged	32,238,388	25,455,920	22,635,032	88.9
$Line7_2$ 21 dpi Control	31,904,354	26,139,229	23,163,003	88.6
$Line7_2$ 21 dpi Challenged	29,734,617	24,212,812	20,912,014	86.4

Table S10. Primers used in ddPCR validation

Primer	Sequence (5’-3’)	Validated in					
		MDV vs Control ($L6_3$)		MDV vs Control ($L7_2$)		$L6_3$ vs $L7_2$ (MDV)	
		10 DPI	21 DPI	10 DPI	21 DPI	10 DPI	21 DPI
ATP6V0D2	F: CAAGAATCCAGCAGAGAGAC	√	√	√	√		
	R: GTCTGCACTAGTCAAGATCC						
CLDN1	F: CAGGGAAGAGGTAGACAGAAAC			√			
	R: CTGGAATGACTCAAGGAGAGAG						
F13A1	F: CCTACATCCCGATCCTCATAG			√	√	√	√
	R: GAATGGAGTTGTTCTCCCTG						
FABP3	F: CCCATAGCACCTTCAAGAAC			√	√		
	R: CATCTAGCTTGACCAAGGAC						
FN1	F: GAGTATACCTACAGCCTCACAG	√					
	R: GCTTCTATCCCAGGAGACTATC						
LAMA1	F: CTCTGGTTCAGATAGTCTCCAG					√	√
	R: CCTGTAGCCTAATGCACATC						
LECT2	F: ATCTCTGGGTCAGGTTACTG			√		√	√
	R: GGTATAGGTCTGAGGAGATGAG						
RGS5	F: GATCTACGAGGAGTTCATCCAG					√	√
	R: CCATCAGAGCAAAGATCCTC						
SERPINB10	F: CTCCTAGAGGGATCCAAAGTTC	√					
	R: GAAAGGAGCAGTAGAGAGACAG						
STAR	F: CCTATCCTTTCCCCTAGTGAAG				√		
	R: GACAAGTTTCACAGGGACTC						
TGM2	F: CTCTGCTGACTGAGTATGAGAC				√		
	R: GATCTCTGGGTTCTGGATGTAG						
WNT7A	F: GAAAGTGGGTAGCAGAGAAG				√		
	R: GGCGAATCCTATTCCGTATC						

Table S11. PCR primers used in ddPCR to examine expression of two MDV microRNAs associated with MDV latency

MDV microRNA	miRNA Sequence	Primer Sequence
miR-M3-3p	UGGGGGGUUCACAUUUUUAAGU	F: GCAGTGGGGGGTTCACATTT
		R: AGCAGGTCCAGTTTTTTTTTTTTTTTACTTAA
miR-M12-3p	UGCAUAAUACGGAGGGUUCU	F: CGCGCAGTGCATAATACGGA
		R: GGTCCAGTTTTTTTTTTTTTTTAGAACCCT
